# Regulation of energy metabolism by combination therapy attenuates cardiac metabolic remodeling in heart failure

**DOI:** 10.7150/ijbs.49520

**Published:** 2020-10-16

**Authors:** Yuting Huang, Kai Zhang, Miaomiao Jiang, Jingyu Ni, Jingrui Chen, Lan Li, Jie Deng, Yan Zhu, Jingyuan Mao, Xiumei Gao, Guanwei Fan

**Affiliations:** 1 First Teaching Hospital of Tianjin University of Traditional Chinese Medicine, Tianjin, People's Republic of China.; 2 State Key Laboratory of Modern Chinese Medicine, Tianjin University of Traditional Chinese Medicine, Tianjin, People's Republic of China.; 3 Tianjin Key Laboratory of Translational Research of TCM Prescription and Syndrome, Tianjin, People's Republic of China.

**Keywords:** cardiovascular systems, energy metabolism, biomarker, multi-omics, combination therapy

## Abstract

Cardiac metabolic remodeling is recognized as an important hallmark of heart failure (HF), while strategies that target energy metabolism have therapeutic potential in treating HF. Shen-Fu formula (S-F) is a standardized herbal preparation frequently used in clinical practice and is a promising combinatorial therapy for HF-related metabolic remodeling. Herein, we performed an untargeted multi-omics analysis using transcriptomics, proteomics, and metabolomics on HF mice induced by transverse aortic constriction (TAC). Integrated and pathway-driven analyses were used to reveal the therapeutic targets associated with S-F treatment. The cardioprotective effect and potential mechanism of S-F were verified by the results from echocardiography, hemodynamics, histopathology, and biochemical assays. As a result, S-F significantly alleviated myocardial fibrosis and hypertrophy, thus reducing the loss of heart function during adverse cardiac remodeling in TAC mice. Integrated omics analysis showed that S-F synergistically mediated the metabolic flexibility of fatty acids and glucose in cardiac energy metabolism. These effects of S-F were confirmed by the activation of AMP-activated protein kinase (AMPK) and its downstream targets in the failing heart. Collectively, our results demonstrated that S-F suppressed cardiac metabolic remodeling through activating AMPK-related pathways via energy-dependent mechanisms.

## Introduction

Heart failure (HF) is a shared chronic stage in several cardiovascular diseases. In China, because of the aging population and the increase in cardiovascular morbidity, the prevalence of HF is rapidly rising and the disease burden remains a challenge 1. According to the 2017 ACC/AHA/HFSA Focused Update Guidelines for the Management of HF, the commonly used treatment for HF includes Class I A recommended ACEI, β-blockers, and aldosterone receptor antagonists 2. However, these drugs do not significantly improve the five-year survival rate of HF patients and may show a ceiling effect. Therefore, there is a need to discover new therapeutic targets and strategies in HF research.

Energy deficiency and myocardial energy metabolism dysfunction are the main signs of HF 3. As early as 2004, Van Bilsen proposed the concept of cardiac metabolic remodeling 4. It occurs mainly due to the switch in cardiac substrates from fatty acids to glucose, which eventually leads to a deficiency in high-energy phosphate and a decrease in energy production. Subsequent studies have shown that changes in cardiac energy metabolism in HF mainly involve energy-starvation, substrate utilization, mitochondrial dysfunction, and insulin resistance 5-8. A direct correlation has been suggested between energy deficit and overall disease severity in HF. Therefore, the modulation of cardiac energy metabolism is an effective way to treat HF. However, since HF is multifactorial, it is difficult to alter the cardiac metabolic network imbalance even with the use of combined single-target drugs. Therefore, combination therapies containing multiple drugs are a new promising approach in the treatment of HF.

Shen-Fu formula (S-F) is a patented herbal preparation consisting of extracts from *Panax Ginseng* and *Radix Aconitum carmichaelii* 9. Previous studies have defined the composition of S-F 10, 11. Over the past 30 years, S-F has been widely used in clinical practice in preventing and treating HF. The active ingredients of S-F are ginsenosides and aconitum alkaloids, including ginsenoside R_1_ (1.15 mg/g), Re (7.68 mg/g), Rg_1_ (3.28 mg/g), Rb_1_ (11.39 mg/g), Rb_2_ (3.64 mg/g), Rb_3_ (0.57 mg/g), Rd (2.11 mg/g), F_2_ (1.45 mg/g), Rg_3_ (0.24 mg/g), as well as benzoylaconine (0.17 mg/g), benzoylhypaconine (0.72 mg/g), and benzoylmesaconine (1.03 mg/g). This herbal preparation has been shown to exhibit a range of pharmacological activities, including potentiating myocardial contractility 12, improving cardiac ejection efficiency 13, promoting antioxidant activities 14, and inhibiting inflammatory response 15. A previous Meta-analysis indicated that S-F combined with routine treatment could significantly improve heart function in HF patients. Besides, S-F showed better effect on clinical effect rate, mortality, heart rate, NT-proBNP and 6-minute walk distance 16. Research using animal models of HF reveals that both ginsenosides and aconitum alkaloids can regulate cardiac energy metabolism dysfunction in ischemic myocardium 17, 18. Our previous study showed that S-F treatment significantly improved cardiac function and alleviated ventricular remodeling in HF mice induced by transverse aortic constriction (TAC). However, the molecular mechanisms of S-F in HF treatment are not well understood.

In this study, multi-omics profiles were integrated with multidimensional data including physiological heart function, hemodynamic assessment, histopathological examination, and mitochondrial function to elucidate the mechanism of S-F on energy metabolism in TAC-induced HF mice. This study established that S-F counteracted cardiac metabolic remodeling by regulating fatty acid and glucose metabolism, and this presents new insights into the energy-dependent mechanism of S-F in the treatment of HF.

## Materials and Methods

### Animals and treatment

Adult male C57 mice (22-24 g, 8 weeks) were purchased from Beijing Vital River Laboratory Animal Technology Co., Ltd. (Certificate number: SCXK [Jing] 2016-0001). Mice were kept in a 12 h dark/light cycle at a temperature of 21 ± 2 °C and 30~70% humidity. They were fed with standard laboratory diet and water. Before surgery, mice were anesthetized using tribromoethanol (1.5%, 1 ml/100 g, i.p.). The mice were subjected to pressure overload through TAC as previously described 19. Briefly, the aortic arch was exposed through an intercostal incision. A 5-0 nylon suture was placed around the aortic arch between the brachiocephalic trunk and the left common carotid artery. A pre-sterilized, blunted 27-gauge needle was then placed within the nylon knot, adjacent to the aorta. After the knot was tightened, the needle was removed from the knot, leaving the aorta permanently constricted. Then the sternum and the skin incision were sutured back in layers with a 5-0 suture. Sham-operated mice received similar surgical procedures but without aortic coarctation. Four weeks after TAC, S-F was administered intragastrically at doses that were equivalent to human doses of 2.5 g, 5 g, and 10 g crude herbs/day, respectively, for 28 days. For the positive control, valsartan was administered by gavage at 10.4 mg/kg. Animal trials were performed in three batches of mice. The total number of animals in each group: sham group (n = 40), TAC group (n = 52), valsartan group (n = 46), 2.5g S-F group (n = 29), 5g S-F group (n = 31), and 10g S-F group (n = 51) (See Supplementary methods for further details). The animal study protocol was approved by the Local Institutional Animal Care and Use Committee (TCM-LAEC20170049) and conformed to the guidelines of the Care and Use of Laboratory Animals published by the Ministry of Science and Technology of China. All experiments complied with the Institutional Animal Care and Use Committee guidelines and were approved by the Animal Care Committee of Tianjin University of Chinese Medicine.

### Echocardiography and hemodynamic measurements

Transthoracic echocardiography was used to detect and record the cardiac function of mice before, and 4 weeks after, S-F was administered. From the long-axis parasternal view, the cardiac cycle was recorded by M-mode ultrasound. Besides, the left ventricle ejection fraction (EF), fractional shortening (FS), interventricular septum thickness at systole (IVS; s) and diastole (IVS; d), left ventricular volume at systole (LV Vol; s) and diastole (LV Vol; d), left ventricular internal systolic (LVID;s) and diastolic (LVID; d) diameters, and left ventricular (LV) mass were measured and analyzed. Hemodynamic characteristics were tested using a 1.4-Fr micro-manometer-tipped catheter (Millar Instruments, Houston, TX, USA) combined with an echocardiography Vevo 2100 Imaging System (VisualSonics, Toronto, ON, Canada).

### Histological and immunofluorescent assessments

Heart samples were excised, washed, and fixed with 4% phosphate-buffered formalin for 48 h. Tissues embedded in wax were cut into 5 mm slices. The sections were stained with hematoxylin and eosin (HE), Masson, and wheat germ agglutinin (WGA) for pathological morphological analysis. The nucleus was localized using 4′,6-diamidino-2-phenylindole (DAPI), and fluorescence images were captured using an inverted fluorescence microscope (ECLIPSE, Nikon, Tokyo, Japan).

### Mitochondrial oxygen consumption rate and ultrastructure

Myocardial mitochondria were extracted using a specialized mitochondrial extraction kit (Solarbio Life Sciences, Beijing, China). The isolated mitochondria were added into the seahorse 24-well plate at the concentration of 40 μg/50 μl ~ 60 μg/50 μl, and centrifuged at 2000 g for 20 min. Oligomycin, carbonyl cyanide 4-trifluoromethoxy phenylhydrazone (FCCP), rotenone/antimycin A were added to the well-incubated probe plate. The mitochondrial respiratory oxygen consumption rate (OCR) was measured at 37 °C using XF^e24^ seahorse cell energy metabolism analyzer (Seahorse Bioscience, Billerica, MA, USA).

The myocardial mitochondria ultrastructure was observed under a transmission electron microscope (TEM) (H-7650, HITACHI, Ibaraki, Japan). The myocardial tissue used for TEM imaging was derived from the left ventricle of the fresh heart. Tissue samples were fixed in 1% OsO_4_ for 30 min, incubated in 4% uranyl acetate, dehydrated in graded ethanol, and embedded in epoxy resin. The sections were double-stained with 4% uranyl acetate and lead citrate.

### Transcriptomics analysis

Total RNA from the mouse heart tissue was extracted using a Trizol reagent kit (Invitrogen, Carlsbad, CA, USA). For quality control, RNA integrity was checked on an Agilent 2100 BioAnalyzer (Agilent Technologies, Santa Clara, CA, USA). Oligo (dT) beads were used to isolate the poly mRNA from the total RNA. The enriched mRNA was fragmented and reverse transcribed into cDNA using random primers. The constructed library was sequenced using an Illumina HiSeq^TM^ 2500 (Illumina, San Diego, CA, USA) by Gene Denovo Biotechnology Co. (Guangzhou, China). Expression of transcripts was quantified using RNA-Seq by expectation-maximization (RSEM) software. Transcripts with an absolute fold-change greater than 1.5 and false detection rate (FDR) below 0.05 were classified as differentially expressed. The differentially expressed genes (DEGs) were further subjected to KEGG pathways enrichment analysis. For details, see Supplementary methods.

### Proteomics analysis

Heart tissues were transferred into a lysis buffer (2% SDS, 7M urea, 1 mg/ml protease inhibitor cocktail), vortexed, and allowed to lyse for 30 min on ice. The samples were homogenized, centrifuged, reduced with TCEP, and alkylated with iodoacetamide. Protein solutions were digested with trypsin and labeled with TMT tags according to the manufacturer's instructions (TMT10plex™ Isobaric Label Reagent Set, Thermo Fisher Scientific, Waltham, MA, USA). The peptide mixture was fractionated using high pH separation on an Ultimate 3000 UPLC system (Thermo Fisher Scientific, Waltham, MA, USA), and further analyzed on an Easy-nLC 1000 system connected to a Q Exactive Hybrid Quadrupole-Orbitrap system (Thermo Fisher Scientific, Waltham, MA, USA). Protein identification was performed using Proteome Discoverer 1.2 (Thermo Fisher Scientific, Waltham, MA, USA) and the Mascot search engine (Matrix Science, London, UK). Proteins with fold changes > 1.2 or < 0.83 and significance level *p* < 0.05 were considered to be differentially expressed. Differentially expressed proteins (DEPs) were further annotated against KEGG databases to determine their functions. For details, see Supplementary methods.

### Metabolomics analysis

Plasma samples (100 μl) were prepared by adding 3 volumes of methanol and 20 μl of internal standard (2-Chloro-L-phenylalanine) to precipitate the proteins. After centrifugation at 13000 g for 15 min, 200 μl of the supernatant was transferred to LC-MS vials. Quality control (QC) samples were prepared by mixing an equal aliquot of the supernatants from each plasma sample. The samples were analyzed using a UHPLC system (1290, Agilent Technologies, Santa Clara, CA, USA) coupled to a Q Exactive Hybrid Quadrupole-Orbitrap system (Thermo Fisher Scientific, Waltham, MA, USA). Data pre-treatment procedures were performed using XCMS software operated in the R computing environment. The processed data files were imported into the SIMCA 14.1 software package (Umetrics, Umeå, Sweden) for multivariate data analysis. Metabolites selected as biomarkers were identified based on the variable importance of project (VIP) threshold of 1 and the two-tailed p-values calculated by student's t-test. Pathway enrichment analysis was performed using the MetaboAnalyst and DAVID toolkits. For details, see Supplementary methods.

### Quantitative real-time PCR (qRT-PCR)

qRT-PCR was performed using a cDNA Reverse Kit and an SYBR Green PCR Master (Roche, Basle, Switzerland) according to the manufacturer's instruction. Semi-quantitative analysis of relative gene expression was performed on a CFX96TM Real-Time PCR Detection System (Bio-Rad, Redmond, WA, USA). GAPDH was used as the housekeeping gene to standardize the amount of mRNA expressed. Details of the 27 mRNAs detected in this study as well as the primer sequences are listed in Table S1.

### Western blot analysis

Heart tissues were homogenized in cold lysis buffer (PMSF: RIPA = 1:100). For western-blotting, the extracted protein was separated on 4-20% SurePAGE^®^ gradient gels (GenScript, Nanjing, China) and then transferred to polyvinylidene fluoride (PVDF) membranes (Thermo Fisher Scientific, Waltham, MA, USA). The membranes were blocked with 5% skim milk powder and incubated with the primary antibody overnight. Immunoblot band intensities were quantified using NIH ImageJ software. Detailed descriptions of the detected proteins can be found in Table S2.

### 18F-FDG PET cardiac glucose metabolism

F-18-fluorodeoxyglucose (18F-FDG) (150 uCi) was injected into the tail vein of mice. The 18F-FDG uptakes of the myocardium were expressed as the standardized uptake value (SUV). The mice underwent a 20-min image acquisition in the prone position 45 min after S-F treatment. 18F-FDG positron emission tomography (PET) coupled with a cardiac PET scanner (Inveon PET/CT, Siemens, Erlangen, Germany) was used to visualize cardiac glucose metabolism 20.

## Results

### S-F exerts a protective effect on myocardial contraction and diastolic function in TAC-induced heart failure mice

**Figure 1:**
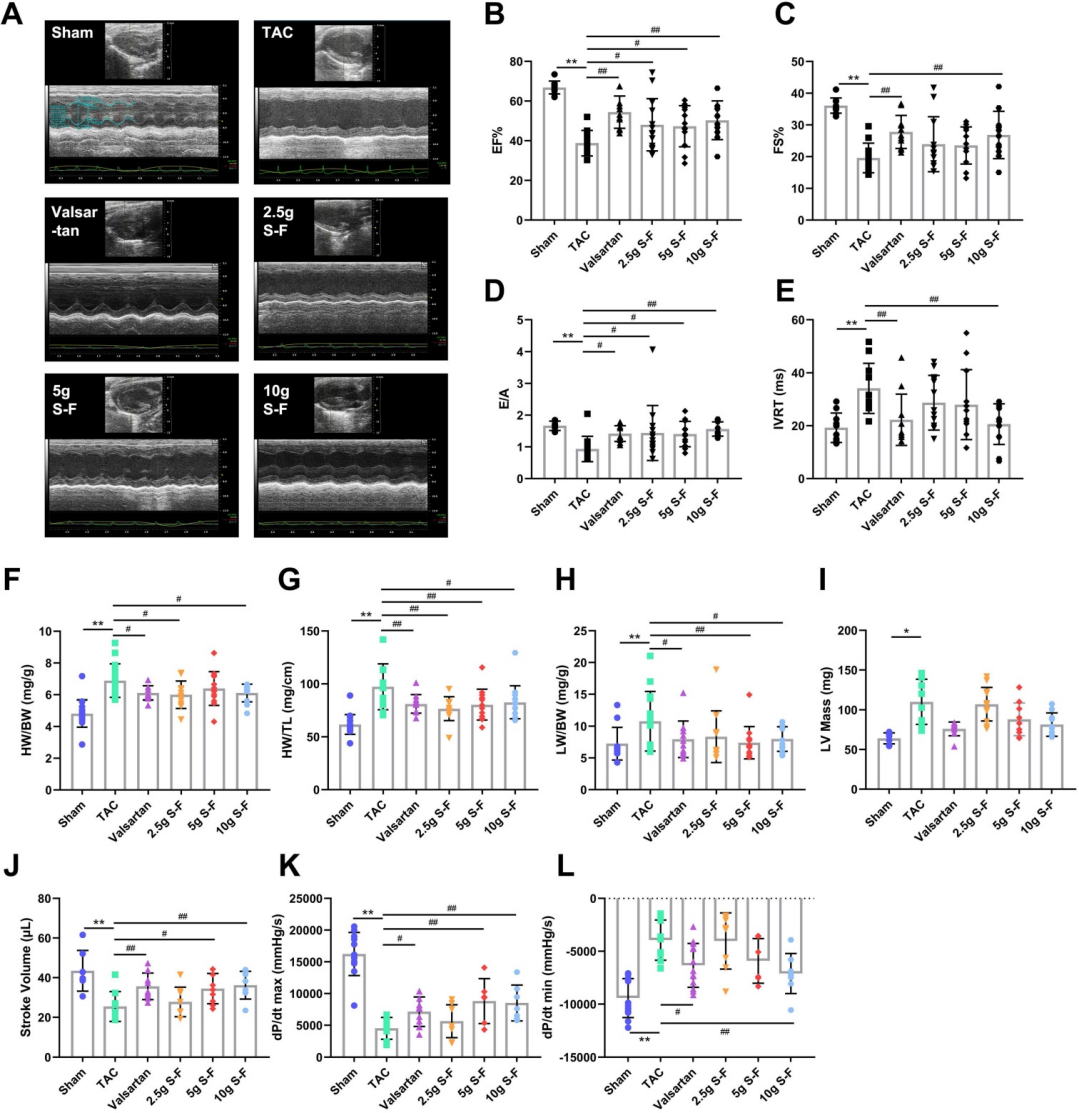
** S-F attenuates transverse aortic constriction (TAC) induced cardiac dysfunction. (A)** Representative M-mode echocardiographic images of mice that underwent the sham operation, TAC only, or TAC followed by treatment with S-F or valsartan.** (B-E)** Echocardiography-derived indices of left ventricle ejection fraction (EF) (**B**), fractional shortening (FS) **(C)**, mitral inflow E and A wave ratio (E/A) **(D)**, and isovolumic relaxation time (IVRT) **(E)**. Heart weight/body weight ratio (HW/BW)** (F)**, heart weight/tibia length ratio (HW/TL)** (G)**, lung weight/body weight ratio (LW/BW)** (H)**, and left ventricular (LV) mass **(I)** were measured after 4 weeks of S-F treatment. **(J-L)** Hemodynamic characteristics assessed by measuring stroke volume (SV) **(J)**, maximum rise/fall rate (dp/dt max)** (K)**, and minimum rise/fall rate (dp/dt min) **(L)** of left ventricular. Data are shown as mean ± SD. *p < 0.05, **p < 0.01, compared with the sham group; ^#^*p* < 0.05, ^##^*p* < 0.01, compared with the TAC group, evaluated by one-way ANOVA with Dunnett's post hoc test.

TAC mice showed significant systolic and diastolic dysfunction and significant decrease in EF and FS, following 8 weeks of modeling (Fig. 1B, C). Besides, there was a significant increase in IVS, LVID and LV Vol (Fig. S1). There were observable changes in the ventricular filling velocity of TAC mice evidenced by decreased mitral inflow E and A wave ratio (E/A) and prolonged isovolumic relaxation time (IVRT) (Fig. 1D, E). Compared with the TAC group, the EF, FS and E/A levels in the valsartan group and S-F groups were significantly increased, while IVRT, IVS, LVID, and LV Vol were significantly decreased.

TAC mice showed a larger heart size compared to hearts of the sham mice. Besides, there was a significant increase in heart weight to body weight (HW/BW) ratio, heart weight to tibia length (HW/TL) ratio, lung weight to body weight (LW/BW) ratio, and LV mass (Fig. 1F-I). S-F and valsartan significantly reduced myocardial enlargement and decreased the values of HW/BW, HW/TL and LW/BW in TAC mice.

Besides, hemodynamic results revealed that S-F and valsartan treatment induced a significant increase in stroke volume (SV), maximum rise/fall rate (dp/dt max), and minimum rise/fall rate (dp/dt min) in TAC mice (Fig. 1J-L), with the effects of 10g S-F being the most significant. These results indicated that S-F significantly counteracted the systolic and diastolic dysfunction, cardiac hypertrophy and ventricular remodeling induced by pressure load.

### S-F alleviates myocardial tissue necrosis, myocardial fibrosis, and cardiac hypertrophy

**Figure 2:**
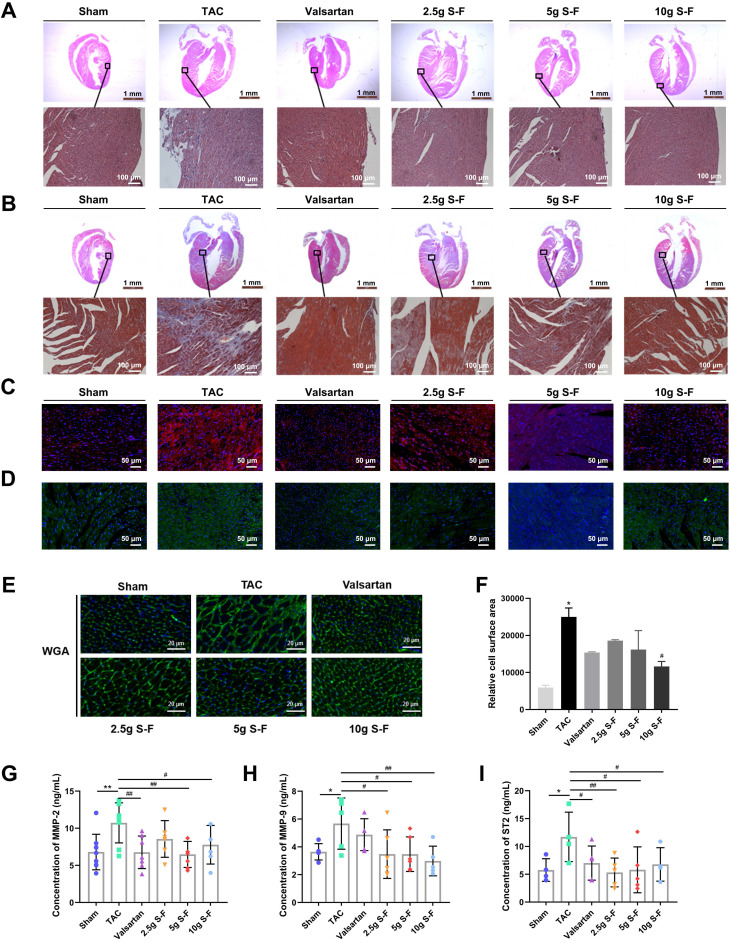
** S-F alleviates myocardial tissue necrosis, myocardial fibrosis, and cardiac hypertrophy. (A, B)** Representative histological images of HE staining results **(A)** and Masson staining **(B)** on heart sections from all the experimental groups. **(C, D)** Representative immunofluorescence images of myocardium sections stained for collagen I **(C)** and collagen III **(D)**. **(E)** Wheat germ agglutinin (WGA) staining followed by **(F)** quantitative analysis of relative cell surface area. **(G-I)** Expression of myocardial extracellular matrix factors MMP-2 **(G)** MMP-9 **(H)** and ST2 **(I)** in plasma was determined by ELISA. Values shown are mean ± SD. *p < 0.05, **p < 0.01, compared with the sham group; ^#^*p* < 0.05, ^##^*p* < 0.01, compared with the TAC group, analysis was performed using one-way ANOVA with Dunnett's post hoc test. MMP, matrix metalloproteinase; ST2, growth stimulation expressed gene 2 protein.

HE staining (Fig. 2A) revealed severe pathological alternations in the myocardium architecture of TAC mice, including myocardial nuclear aggregation, disordered cell arrangement, and diffuse edema compared with that of sham mice. After 4 weeks of treatment, the S-F and valsartan groups showed alleviated myocardial necrosis compared with the model group. As shown in Fig. 2B, the myocardial cells in TAC mice were disorderly arranged, the area of collagen fibers and the degree of myocardial fibrosis was significantly increased. Moreover, TAC mice showed increased staining of collagen I and III when compared to sham mice (Fig. 2C, D). S-F treatment reduced the collagen fiber area, and the myocardial cells were structurally intact and neatly arranged. WGA staining (Fig. 2E) showed that the myocardial cell gap widened, the cell membrane disappeared, and the area of myocardial cells increased significantly in TAC mice compared with sham mice. In contrast, S-F treatment narrowed the myocardial cell gap, the cell membrane was relatively intact, and the myocardial cell area was significantly reduced (Fig. 2F).

The plasma levels of myocardial extracellular matrix factors (MMP) and the expression of genes related to myocardial fibrosis and cardiac hypertrophy was determined. TAC mice showed increased levels of MMP-2, MMP-9, and ST2 in plasma (Fig. 2G-I), increased mRNA expression of atrial natriuretic peptide (ANP), brain natriuretic peptide (BNP), collagen I, fibronectin, β-myosin heavy chain (β-MHC) in myocardial tissue, and decreased expression of smooth muscle actin (SMA) and α-myosin heavy chain (α-MHC) (Fig. S2), when compared with sham mice. S-F showed a regulatory effect on the above abnormal indicators, suggesting that it decreases the degree of myocardial tissue necrosis, myocardial fibrosis, and cardiac hypertrophy, thereby improving cardiac pathology.

### S-F improves the oxygen consumption rate (OCR) and enhances the structural and functional integrity of myocardial mitochondria

**Figure 3:**
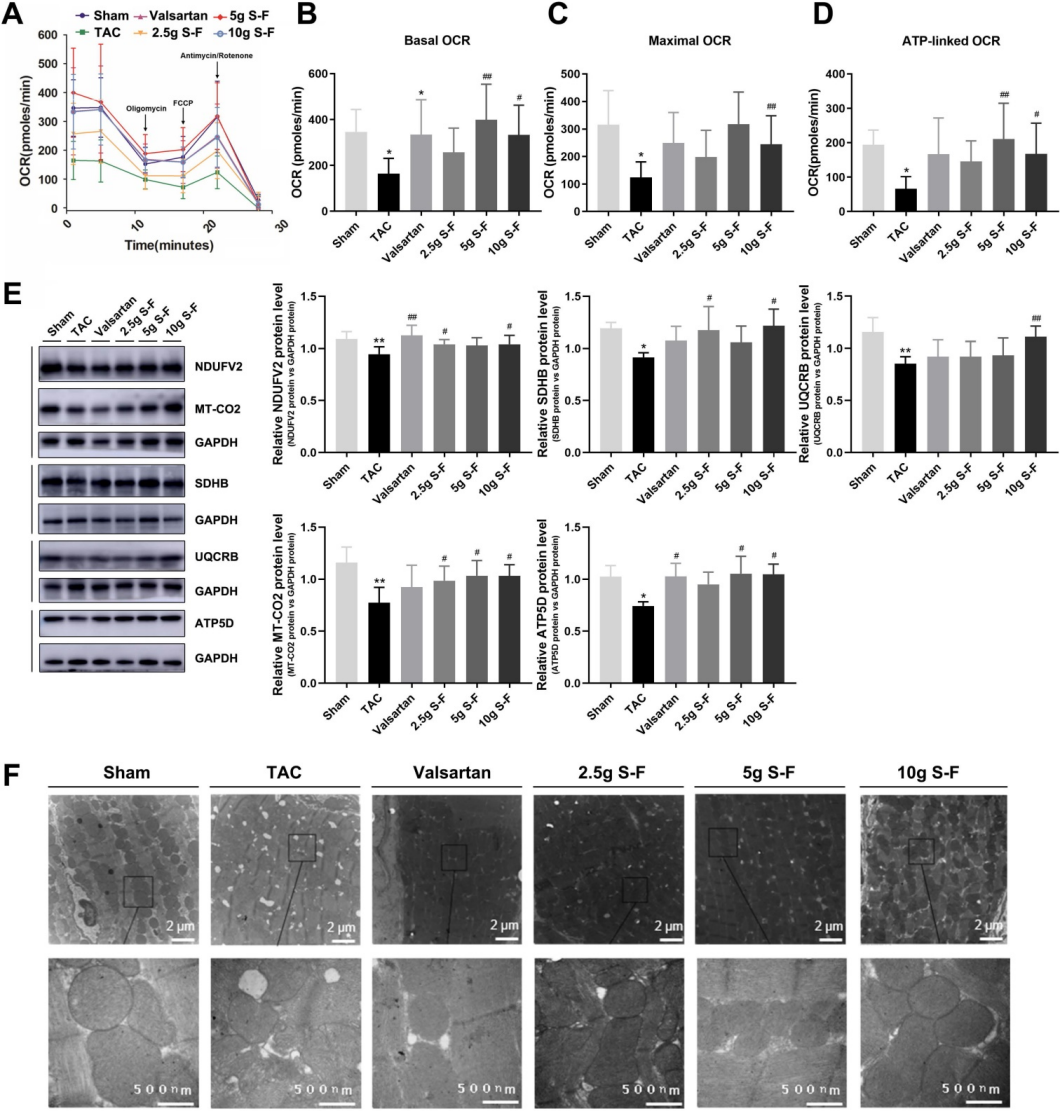
** S-F enhances mitochondrial oxygen consumption rate (OCR) and maintains mitochondrial structure. (A)** OCR curves of myocardial mitochondria. Oligomycin, carbonyl cyanide 4-trifluoromethoxy phenylhydrazone (FCCP), rotenone and antimycin A were injected sequentially at the indicated time points into each well containing myocardial mitochondria. Mitochondrial function was assessed by the basal OCR **(B)**, maximal OCR **(C)**, and ATP-linked OCR **(D)**. **(E)** Western blot analysis was performed to determine expression of mitochondrial complex I (NADH dehydrogenase ubiquinone flavoprotein 2, NDUFV2), complex II (succinate dehydrogenase subunit B, SDHB), complex III (ubiquinol-cytochrome C reductase binding protein, UQCRB), complex IV (cytochrome C oxidase subunit II, MT-CO2), and complex V (ATP synthase subunit delta, ATP5D). Values shown are mean ± SD. *p < 0.05, **p < 0.01, compared with the sham group; ^#^*p* < 0.05, ^##^*p* < 0.01, compared with the TAC group, evaluated by one-way ANOVA with Dunnett's post hoc test. **(F)** Representative transmission electron microscopy images showing the mitochondrial network in myocardial tissue in all experimental groups.

TAC mice showed a significant decline in basal, maximal and ATP-linked OCRs compared to sham mice (Fig. 3A). S-F treatment resulted in enhanced OCRs in basal (Fig. 3B), maximal (Fig. 3C) and ATP-linked respiration (Fig. 3D). To further investigate the effect of S-F on mitochondrial function, the protein and mRNA expression levels of five mitochondrial complexes were measured. The expression of complexes I, II, III, IV and V (Fig. 3E and Fig. S3) were significantly decreased in TAC mice, and these abnormities were significantly restored by S-F treatment. In comparison, valsartan only had a significant effect on the basal OCR and the levels of complex I and V.

Ultrastructural aspects of the mitochondria in myocardium cells revealed structural disorders with vacuolar degeneration and edematous myocardial fibers in the TAC group (Fig. 3F). Upon treatment with 10g S-F, the mitochondria were neatly arranged, there was a reduction in the mitochondrial edema, and the mitochondria structure became relatively clear and intact. These results indicated that S-F played an anti-HF effect by improving the mitochondrial OCRs, regulating mitochondrial function and structure, and alleviating mitochondrial energy metabolism dysfunction.

### S-F mediates abnormalities in energy metabolic pathways at the transcript, protein, and metabolite levels in TAC mice

**Figure 4:**
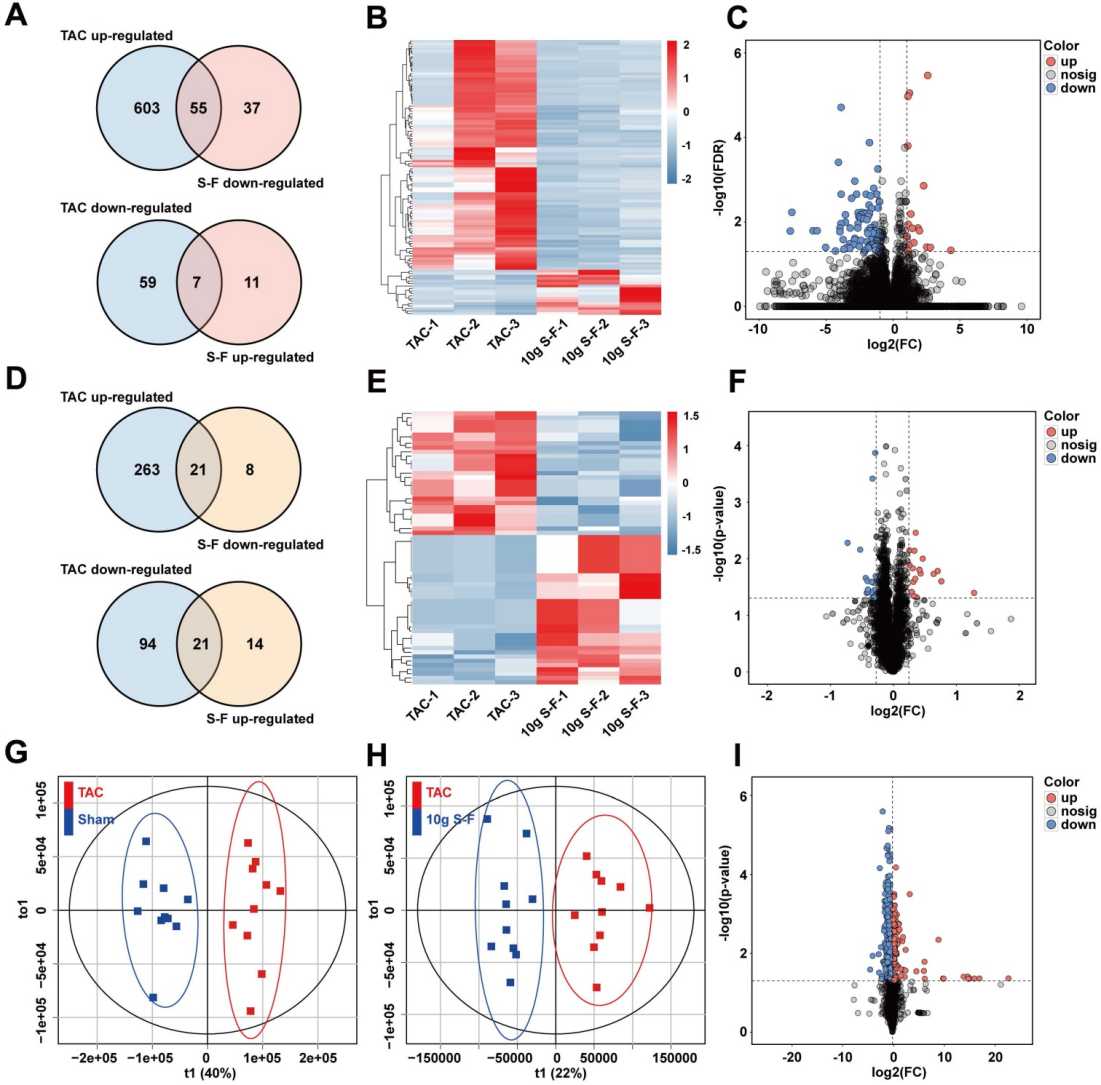
** Signatures of S-F treatment at transcript, protein, and metabolite levels. (A)** Venn-diagrams showing the number of differentially expressed genes (DEGs) that were up-regulated in the TAC group (compared with the sham group) and down-regulated by S-F (compared with the TAC group), and DEGs that were down-regulated in TAC mice and up-regulated by S-F. **(B)** Heatmap representation of hierarchical clustering of DEGs that were significantly reversed by S-F treatment. **(C)** Volcano plot showing the distribution of DEGs in the 10g S-F group compared to the TAC group. **(D)** Venn-diagrams showing the number of differentially expressed proteins (DEPs) in TAC mice compared to sham mice, and DEPs in S-F treated mice compared to TAC ones. **(E)** Heatmap of DEPs that were significantly reversed by S-F treatment. **(F)** Volcano plot of DEPs in the 10g S-F group compared to the TAC group. **(G)** Plot of OPLS-DA scores derived from plasma metabolic profiling of the TAC and sham groups (negative ion detection mode). **(H)** Plot of OPLS-DA scores of the 10g S-F group versus those of the TAC group (negative ion mode). **(I)** Volcano plot showing the distribution of differentially expressed metabolites in the 10g S-F group compared to the TAC group. The number of animals: transcriptomics analysis (n = 3 in each group), proteomics analysis (n = 3 in each group), metabolomics analysis (n = 10 in each group). OPLS-DA, orthogonal partial least squares discriminant analysis.

Untargeted multi-omics analysis using transcriptomics, proteomics, and metabolomics on the sham, TAC, and 10g S-F treated mice were performed to obtain a comprehensive view of the complex mechanisms of S-F. A total of 772 genes were identified as differentially expressed genes (DEGs) at the transcriptome level, out of which 724 were altered in TAC mice relative to sham mice, while 110 DEGs were restored through S-F treatment (Fig. 4A). Principal component analysis (PCA) of transcriptome data showed that samples from the TAC group were distinct from those in the sham and S-F group (Fig. S4A). Upon comparing the S-F group with the TAC model, 92 DEGs were found to be significantly down-regulated, while 18 DEGs were significantly up-regulated. These results were visualized in the hierarchically clustered heatmap (Fig. 4B) and volcano plot (Fig. 4C). At the proteome level, a total of 421 proteins were differentially expressed. The Venn diagram in Fig. 4D shows the number of differentially expressed proteins (DEPs) in the TAC group compared to the S-F group. Among the 64 proteins that were significantly affected by S-F, 29 were down-regulated and 35 were up-regulated. The corresponding heatmap and volcano plot are shown in Fig. 4E, F. Besides, the metabolic profiles of the plasma samples collected from the three groups are shown in the PCA scores plot (Fig. S4D, E). Accurate discrimination between sham and TAC groups were attained using orthogonal partial least squares discriminant analysis (OPLS-DA) (Fig. 4G, 2-component model, R^2^X=0.59, R^2^Y=0.93, Q^2^Y=0.80). Mice in the S-F group were distinct from those in the TAC group, evidenced by the robust OPLS-DA model (Fig. 4H, 2-component model, R^2^X=0.56, R^2^Y=0.89, Q^2^Y=0.50). A total of 721 metabolites were found to be differentially expressed metabolites (DEMs) in the positive and negative ion modes (Fig. S4F). A total of 336 DEMs were down-regulated and 117 DEMs were up-regulated after S-F treatment (Fig. 4I).

**Figure 5:**
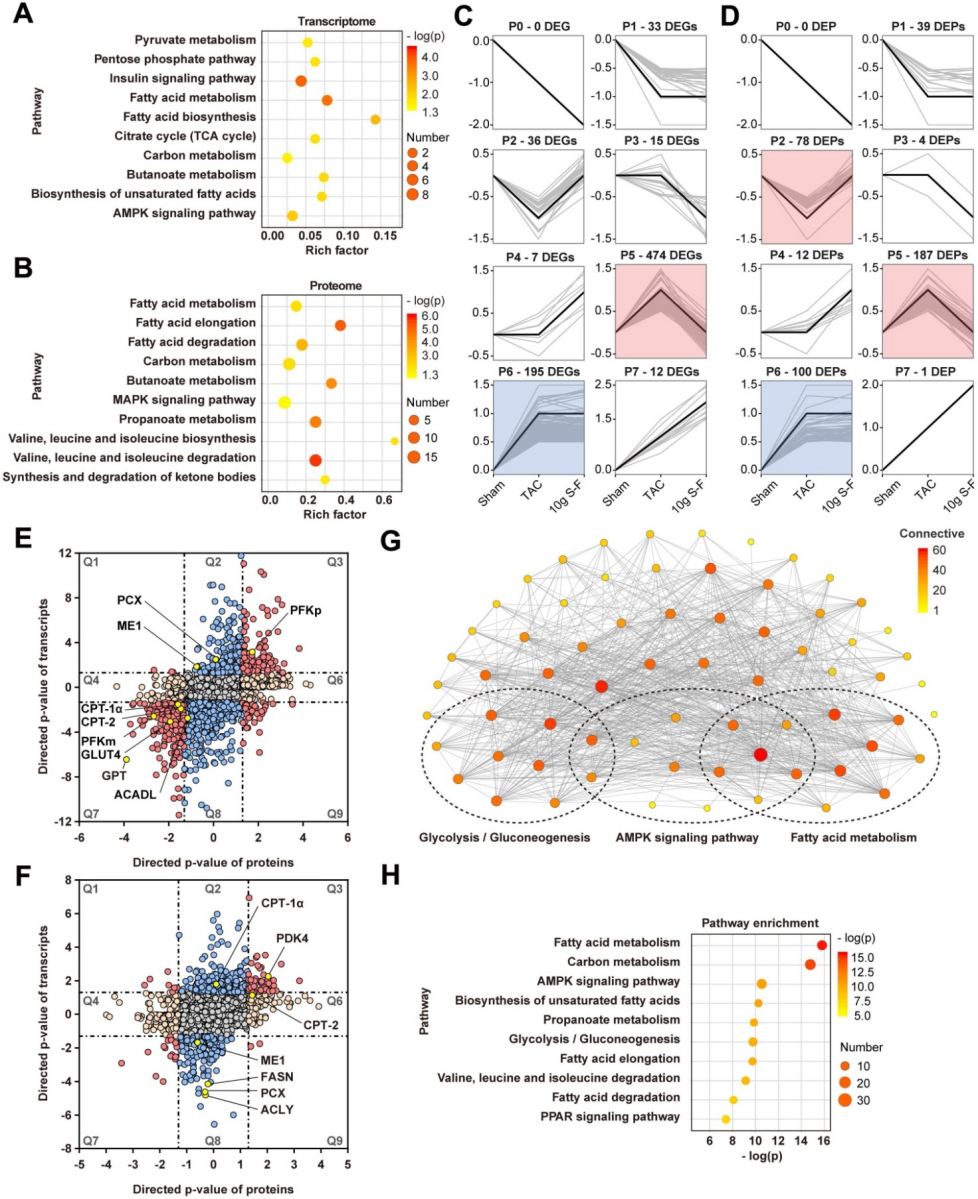
** Network analysis of transcriptome and proteome. (A, B)** KEGG pathway enrichment analysis of DEGs **(A)** and DEPs **(B)**. Rich factor refers to the ratio of the number of DEGs (or DEPs) in the pathway entry to the total number of genes (or proteins) in the pathway entry. Pathways with p-value < 0.05 were considered significantly enriched. **(C, D)** Short time-series expression miner (STEM) clustering on DEGs **(C)** and DEPs **(D)**. Each square represents expression trend of gene (or protein) in the sham, TAC and 10g S-F groups. The black polygonal line in the square indicates the overall expression profile of all DEG (or DEP) in the corresponding set. The profile ID and the number of DEGs and DEPs in each profile are shown on the top of the square. Colored profiles are those that are statistically significant (p < 0.05). **(E)** Combined analysis of DEGs and DEPs in TAC mice relative to sham mice. **(F)** Combined analysis of DEGs and DEPs in S-F treated mice relative to TAC ones. The omics datasets were merged based on the gene symbol. Directed p-values are defined as -log_10_ (p-value) times the direction of the effect. Red dots in the Q3 or Q7 quadrants indicate co-regulated entities that were consistently up-regulated or down-regulated. Blue dots in the Q2 and Q8 quadrants represent genes with significant changes in the transcriptome, while orange dots in the Q4 and Q6 quadrants represent significant genes in the proteome. **(G)** Interaction networks of 68 DEGs and DEPs involved in energy metabolism. Within networks, nodes are colored according to the number of connections. **(H)** Pathway enrichment analysis of the above DEGs and DEPs ranked by the -log_10_ (p-value). DEGs, differentially expressed genes; DEPs, differentially expressed proteins.

To evaluate the potential function of DEGs and DEPs, pathway enrichment analysis based on the KEGG database was performed. As shown in Fig. 5A, B, both DEGs and DEPs were significantly enriched in pathways related to energy metabolism, such as fatty acid metabolism and carbohydrate metabolism. Interestingly, the DEGs and DEPs involved in energy metabolic pathway were mainly distributed in the expression patterns of profiles 5 and 2 in short time-series expression miner (STEM) analysis (Fig. 5C, D). This suggested that S-F had a significant regulatory effect on energy metabolism in HF mice.

**Figure 6:**
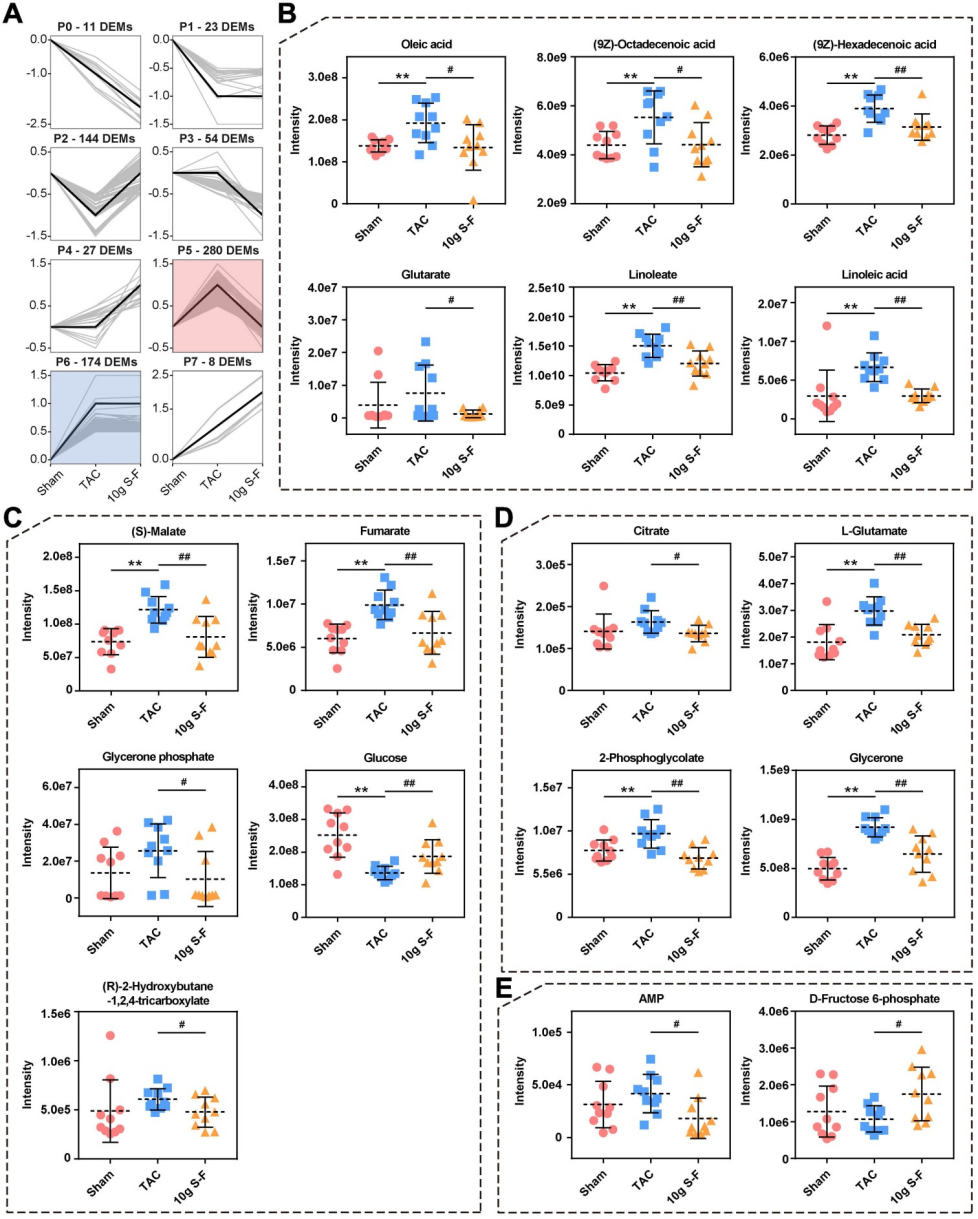
** Alterations in metabolome in response to S-F treatment. (A)** STEM clustering on differentially expressed metabolites in the sham, TAC and 10g S-F groups. **(B-E)** Significantly altered metabolites in the energy metabolic pathways related to S-F treatment, including oleic acid, (9Z)-octadecenoic acid, (9Z)-hexadecenoic acid, glutarate, linoleate, and linoleic acid in the fatty acid metabolic pathways **(B)**, (S)-malate, fumarate, glycerone phosphate, glucose, and (R)-2-hydroxybutane-1,2,4-tricarboxylate in the pyruvate and glucose metabolic pathways **(C)**, citrate, L-glutamate, 2-phosphoglycolate, and glycerone in carbon metabolism **(D)**, and AMP, D-fructose 6-phosphate in the AMPK signaling pathway **(E)**. Values shown are mean ± SD. *p < 0.05, **p < 0.01, compared with the sham group; ^#^*p* < 0.05, ^##^*p* < 0.01, compared with the TAC group.

Furthermore, the transcriptome and proteome data were combined to identify genes that were altered in both omics layers. A total of 3418 genes were found to be co-regulated genes in TAC mice, out of which 96 were up-regulated (Fig. 5E, Q3) and 200 were down-regulated (Fig. 5E, Q7). After S-F treatment, a total of 63 genes differed significantly in protein abundance and transcript status (Fig. 5F). Significantly differentially expressed genes of energy metabolism in transcriptomics and proteomics included CPT-1α, CPT-2, ACADL, and FASN, which are involved in fatty acid metabolism; and ME1, PCX, PDK4, PFKm, and GLUT4, which are related to pyruvate and glucose metabolism. Moreover, an interaction network between 68 mRNAs and proteins involved in the energy metabolic pathway was established (Fig. 5G). They were found to be significantly enriched in fatty acid and glucose metabolism, as well as AMP-activated protein kinase (AMPK) and peroxisome proliferator-activated receptor (PPAR) signaling pathways (Fig. 5H). Meanwhile, metabolomics studies showed significant changes in the level of key metabolites related to energy metabolism, such as oleic acid, (9Z)-octadecenoic acid, (9Z)-hexadecenoic acid, glutarate, linoleate, and linoleic acid in the fatty acid metabolic pathways (Fig. 6B), (S)-malate, fumarate, glucose, and glycerone phosphate in the glucose metabolic pathways (Fig. 6C), as well as AMP and D-fructose 6-phosphate in the AMPK signaling pathway (Fig. 6E). Similarly, the significantly altered metabolites were mainly concentrated on profile 5 in STEM analysis (Fig. 6A), thus revealing the significant effect of S-F on energy metabolism at the downstream metabolic status. These results suggested that S-F caused synergistic effects on energy metabolism in HF mice at the transcript, protein, and metabolite levels. Fatty acid metabolism and glucose metabolism were identified as the most influenced metabolic pathways associated with S-F treatment.

### Validation of the regulatory effect of S-F on glucose and fatty acid metabolism

**Figure 7:**
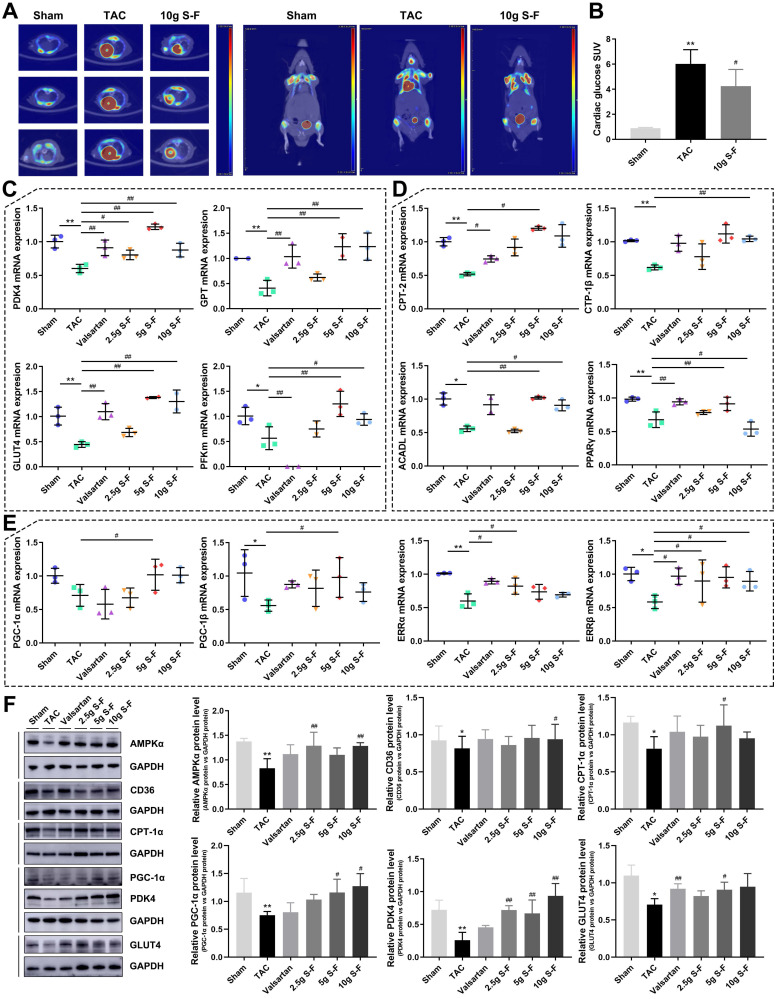
** Validation of the effect of S-F on glucose and fatty acid metabolism in TAC mice. (A)** Differential F-18-fluorodeoxyglucose (18F-FDG) positron emission tomography (PET) uptake with PET/CT in cardiac muscle of mice. High signal density is colored in red, and low signal density is colored in blue and green. **(B)** Quantitative analysis of myocardial glucose uptake based on standardized uptake values (SUVs). Signals were measured from three biological replicates, and three technical repeats for each replicate. **(C)** qRT-PCR analysis of key genes that regulate glucose metabolism in heart tissues, including pyruvate dehydrogenase kinase 4 (PDK4), pyruvate/alanine aminotransferases (GPT), glucose transporter 4 (GLUT4), and phosphofructokinase muscle (PFKm). **(D)** qRT-PCR analysis of mRNA levels of genes that regulate fatty acid oxidation, including carnitine palmitoyl transferase II (CPT-2), carnitine palmitoyl transferase-1β (CPT-1β), long-chain acyl-CoA dehydrogenase (ACADL), and peroxisome proliferator-activated receptor γ (PPARγ). **(E)** mRNA levels of PPARγ coactivators (PGC-1α and PGC-1β), and estrogen-related receptors (ERRα and ERRβ). **(F)** Western blot analysis of the expression of AMP-activated protein kinase α (AMPKα), fatty acid translocase (CD36), CPT-1α, PGC-1α, PDK4 and GLUT4. Data are shown as mean ± SD. *p < 0.05, **p < 0.01, compared with the sham group; ^#^*p* < 0.05, ^##^*p* < 0.01, compared with the TAC group, evaluated by one-way ANOVA with Dunnett's post hoc test.

The 18F-FDG PET method was used to compare the cardiac glucose uptake of mice in the sham group, model group and 10g S-F group (Fig. 7A). In the TAC group, the mean glucose uptake SUV significantly increased in the 8^th^ week compared with that in the sham group (6.03 vs 0.87). However, S-F was found to significantly restore the abnormal glucose uptake in the failing heart 4 weeks after treatment (Fig. 7B).

The key transcription factors related to glucose and fatty acid metabolism were measured to establish the potential effects of S-F in the energy remodeling of HF. As shown in Fig. 7C and Fig. S5, the expression of pyruvate dehydrogenase kinase 4 (PDK4), pyruvate/alanine aminotransferases (GPT), transcripts encoding glucose transporters (GLUT4), phosphofructokinase muscle (PFKm), pyruvate carboxylase (PCX), and monocarboxylate pyruvate transporter (MCT1) were found to be reduced in the failing heart. These alterations confirmed impairment in pyruvate transport, glucose uptake and metabolism. Consistent with the decreased expression of transcription factors in glucose metabolism, mRNA levels of key enzymes of fatty acid oxidation (Fig. 7D) including carnitine palmitoyl transferase II (CPT-2), carnitine palmitoyl transferase-1β (CPT-1β), long-chain acyl-CoA dehydrogenase (ACADL), and PPARγ were significantly decreased in TAC mice. Meanwhile, the transcript levels of PPARγ coactivator 1 (PGC-1α and PGC-1β), two master regulators of mitochondrial biogenesis and metabolism, and estrogen-related receptors (ERRα and ERRβ) were reduced in TAC mice (Fig. 7E).

S-F treatment significantly affected the metabolic transcripts associated with glucose and fatty acid metabolism. The expression of key genes in glucose metabolism and fatty acid oxidation including PDK4, GPT, GLUT4, PFKm, PCX, CPT-2, CPT-1β, ACADL, and PPARγ were up-regulated in varying degrees following S-F treatment (Fig. 7C, D). The expression of key mitochondrial transcription factor coactivators, such as PGC-1α, PGC-1β, ERRα and ERRβ, was also up-regulated after S-F treatment (Fig. 7E). These results indicated that S-F synergistically mediated the abnormalities in glucose and fatty acid metabolic pathways in TAC mice.

Western blot analysis was performed to detect the protein levels of AMPKα, CD36, CPT-1α, PGC-1α, PDK4, GLUT4, and PPARα (see Fig. 7F and Fig. S5). The expression of these proteins was reported to be down-regulated in TAC mice and was significantly restored by S-F treatment, but the effect of valsartan treatment was not significant. Among them, the AMPK-related signaling pathway was most significantly affected by S-F treatment.

## Discussion

Pressure overload triggers complex biological responses that result in HF which is characterized by cardiac remodeling 21, 22. In the current study, remarkable cardiac remodeling and heart dysfunction occurred in TAC mice, evidenced by significantly increased cardiac fibrosis and hypertrophy, as well as diminished echocardiographic features. S-F and valsartan treatment exhibited a protective function against myocardial fibrosis and necrosis which contributed to cardiac remodeling. Furthermore, both S-F and valsartan effectively counteracted the systolic and diastolic dysfunction, improved cardiac hemodynamics, reduced myocardial enlargement, and even down-regulated the levels of extracellular matrix markers in TAC mice. These findings indicated significant improvement in heart function and inhibition of cardiac remodeling in TAC mice after S-F treatment, and this supported the therapeutic value of S-F in HF. Comparatively, the main advantage of S-F over the positive control valsartan is at the regulation of energy metabolism as well as mitochondrial function.

Mounting evidence suggests that impaired energy metabolism and mitochondrial function contribute to cardiac remodeling leading to HF 23. The efficiency of oxygen utilization is relatively low during HF, exhibiting an energy deficit (containing 30-40% less ATP compared with a healthy heart) due to changes in energy substrate and a gradual decline in mitochondrial oxidative phosphorylation 24. In mouse hearts with systolic dysfunction induced by TAC, reduced mitochondrial OCRs and abnormal mitochondrial morphology were observed. Decreased mitochondrial respiration due to downregulation of mitochondrial complexes may be an important factor in linking contractile function and energy metabolism to the progression of HF 25. S-F was found to significantly ameliorate mitochondrial dysfunction by enhancing OCRs and maintaining the mitochondrial structure. This suggests that the regulation of mitochondrial energy metabolism is one of the potential mechanisms of S-F action.

Alterations in energy substrate metabolism are recognized as important hallmarks in the cardiac remodeling process 26. Approximately 70% to 90% of cardiac ATP originates from fatty acids metabolism. The remaining 10% to 30% comes from glucose and lactate metabolism, as well as small amounts of ketones and amino acids 3, 27. During cardiac metabolic remodeling, energy metabolism is reprogrammed toward increased utilization of glucose and with significant downregulation of fatty acid oxidation 28. Even though glucose metabolism consumes less oxygen, it produces fewer amounts of energy compared with fatty acid oxidation, causing the heart to remain in an energy-starved state 24. Recent evidence suggests that maintaining fatty acid oxidation during pressure overload prevents increased dependence on glucose and further suppresses the effects of glucose on pathological growth 29, 30. Although the mechanistic link between fatty acid metabolism and contractile function remains controversial, promoting fatty acid utilization has been shown to be a potential therapeutic approach towards decreasing the severity of HF 29, 31, 32. In this study, abnormalities in key genes involved in fatty acid oxidation and transport were significantly restored after S-F treatment. This regulatory effect of S-F on fatty acid utilization was also confirmed through the multi-omics study. Integrated and pathway-driven analysis identified that fatty acid metabolism was the major pathway associated with S-F. Besides, the downstream metabolome showed a significant decrease in the plasma levels of the free fatty acids in TAC mice after S-F treatment.

Besides fatty acid utilization, S-F was found to have a regulatory effect on glucose metabolism. In the presence of systolic dysfunction, cardiac glucose uptake was found to be increased in TAC rats. Similar findings have been reported in previous studies 33, 34. While glucose uptake into cardiac myocytes is increased, their subsequent entry into the mitochondrial is decreased. This results in a marked reduction in glucose oxidation and an increase in glycolysis 6. The impaired glucose metabolism that parallels systolic dysfunction might be due in part to mitochondrial dysfunction or reduced expression of GPT, PFKm, PDK4, and GLUT4 involved in glucose oxidation. Another factor that may influence glucose oxidation is the fact that pyruvate may be channeled into anaplerotic pathways 35. Higher levels of ME1 in mice hearts subjected to TAC and elevated abundance of (S)-malate and fumarate in mice plasma were reported. Increased anaplerotic flux via pyruvate carboxylation by ME1 may lead to a decrease in oxidation, which reduces the efficiency of ATP synthesis and exacerbates pathological remodeling 36, 37. After 4 weeks of S-F treatment, the plasma levels of (S)-malate in TAC mice was significantly decreased, the transcript levels of ME1 were down-regulated, and the expression of PDK4, GPT, GLUT4, and PFKm was up-regulated. These alterations were accompanied by a reduction in cardiac glucose uptake in the S-F treated mice. Taken together, these results suggest that S-F may regulate the balance in glucose and fatty acids utilization, thus maintaining metabolic flexibility in energy metabolism.

AMPK pathway is one of the key signaling pathways that responded to S-F treatment. Activation of AMPK in HF may induce a wide range of effects that coordinately improve cardiac function and alleviate metabolic remodeling 38. Specifically, increased AMPK activity could stimulate both fatty acid and glucose utilization, thereby restoring energy supply 39. On the one hand, AMPK can stimulate fatty acids entry into the mitochondria and subsequent oxidation 40. This increases the translocation of fatty acid translocase CD36 to the sarcolemma, promoting cardiomyocyte uptake of fatty acids 41. Activated AMPK further inhibits the production of malonyl-CoA and activates CPT-1, while CPT-1 controls the rate-limiting step of mitochondrial fatty acid β-oxidation 42. On the other hand, AMPK mediates the translocation of GLUT4 from the cytosol to the membrane and phosphorylates phosphofructokinase, thereby promoting glucose utilization as a cardioprotective response 43. Furthermore, AMPK may also improve mitochondrial biogenesis in a long-term manner by activating PGC-1α 44. PGC-1α further binds to and coactivates PPAR and ERR leading to increased fatty acid uptake and oxidation 45. This study showed that S-F activated the AMPK signaling pathway and significantly up-regulated the levels of CD36, CPT-1α, PGC-1α, PPARα, ERRα, ERRβ, GLUT4 and PFKm in TAC mice. These results were validated in omics studies, western-blot analysis and qRT-PCR results, suggesting that S-F synergistically mediated the metabolic flexibility of fatty acids and glucose in cardiac energy metabolism, with AMPK pathway being the most impacted pathway.

This study was not without limitations. First, although S-F can modulate glucose uptake and utilization, this mechanism needs to be further explored. A significant increase in GLUT4 expression was observed in S-F treated mice, however, GLUT1 showed no significant change (data not shown). This was inconsistent with the reduction in cardiac glucose uptake and was attributed to the integrated effects of multiple components in S-F. Second, energy metabolic pathways that were the most relevant to the synergistic effects of S-F treatment were validated. Integrated omics analysis showed that S-F exerted an effect on branched chain amino acids and insulin-related pathways; however, this effect requires to be studied further. Finally, the relatively small-scale sample sets of omics studies could have possibly introduced selection bias. Therefore, large-sample sets examining the potential markers associated with S-F treatment should be conducted.

## Conclusion

In conclusion, the key strength of this study is in its comprehensive design which reveals the energy-dependent mechanism of S-F through global multi-omics profiling. This study demonstrates that S-F can improve cardiac function, ameliorate mitochondrial dysfunction, and further alleviate metabolic remodeling in the failing heart. This is exhibited in the integrated omics analysis, which shows that S-F causes synergistic effects on fatty acids and glucose metabolism. The potential mechanism underlying these effects may be related to the regulation of AMPK-related signaling pathways. These findings provide new insights into the mechanism of S-F and provide evidence for the cardioprotective effect of S-F in the treatment of HF.

## Supplementary Material

Supplementary figures and tables.

## Abbreviations

HF: heart failure
S-F: Shen-Fu formula
TAC: transverse aortic constriction
EF: ejection fraction
FS: fractional shortening
IVS: interventricular septum thickness
LV Vol: left ventricular volume
LVID: left ventricular internal diameters
OCR: oxygen consumption rate
VIP: variable importance of project
18F-FDG: F-18-fluorodeoxyglucose
SUV: standardized uptake value
PET: positron emission tomography
E/A: E and A wave ratio
IVRT: isovolumic relaxation time
HW/BW: heart weight to body weight ratio
HW/TL: heart weight to tibia length ratio
LW/BW: lung weight to body weight ratio
SV: stroke volume
dp/dt max: maximum rise/fall rate
dp/dt min: minimum rise/fall rate
MMP: matrix metalloproteinase
ST2: growth stimulation expressed gene 2 protein
ANP: atrial natriuretic peptide
BNP: brain natriuretic peptide
MHC: myosin heavy chain
SMA: smooth muscle actin
PCA: principal component analysis
OPLS-DA: orthogonal partial least squares discriminant analysis
DEGs: differentially expressed genes
DEPs: differentially expressed proteins
DEMs: differentially expressed metabolites
STEM: short time-series expression miner
AMPK: AMP-activated protein kinase
PPAR: peroxisome proliferator-activated receptor
PGC: PPARγ coactivator
GPT: pyruvate/alanine aminotransferases
PDK4: pyruvate dehydrogenase kinase 4
GLUT4: transcripts encoding glucose transporter 4
PFKm: phosphofructokinase muscle
PCX: pyruvate carboxylase
MCT: monocarboxylate pyruvate transporter
CPT: carnitine palmitoyl transferase
ACADL: long-chain acyl-CoA dehydrogenase
ERR: estrogen-related receptor
