# Supplementary material for: Regulation of energy metabolism by combination therapy attenuates cardiac metabolic remodeling in heart failure
Source: Int J Biol Sci. 2020 Oct 16;16(16):3133–48. doi: 10.7150/ijbs.49520 (PMC7645995; doi:10.7150/ijbs.49520)
Supplement: Supplementary file 1 — Supplementary figures and tables. [file ijbsv16p3133s1.pdf]

Supplementary materials

Table of contents

Supplementary results……………………………………………………………………… 2

Table S1 Primers sequences used for real-time PCR. ………………………………………… 2

Table S2 Detailed descriptions of the detected proteins. ……………………………………… 4

Figure S1 Bar plots showing IVS, LVID, and LV Vol evaluated by echocardiography in sham, TAC, and S-F treated mice. ………………………………………………………………………. 5

Figure S2 qRT-PCR analysis of mRNA levels of the heart failure related factors ANP, BNP, collagen I, fibronectin, SMA, α-MHC, and β-MHC…………………………………………….. 6

Figure S3 qRT-PCR analysis of mRNA levels of mitochondrial complexes in heart tissues.… 7

Figure S4 Transcriptomics, proteomics, and metabolomics analysis. ………………………… 8

Figure S5 mRNA levels of PCX, MCT1, and protein expression of PPARα in heart tissues. …………………………………………………………………………………………… 10

Supplementary methods…………………………………………………………………… 11

Experimental animal grouping. ……………………………………………………………... 11

Transcriptomics analysis. ……………………………………………………………………… 11

Proteomics analysis. …………………………………………………………………………… 12

Metabolomics analysis.………………………………………………………………………… 14

Supplementary results

Table S1 Primers sequences used for real-time PCR.

mRNA (mouse)		Sequence
Collagen I	Forward	TCGAGGTCGCACTGGCGATAGTGG
	Reverse	GCCTCGGTGGACATCAGGCGC
α-MHC	Forward	ATAAAGGGGCTGGAGCACTG
	Reverse	GCCTCTAGGCGTTCCTTCTC
ANP	Forward	AAGAACCTGCTAGACCACCTGGAG
	Reverse	TGCTTCCTCAGTCTGCTCACTCAG
BNP	Forward	GGAAGTCCTAGCCAGTCTCCAGAG
	Reverse	GCCTTGGTCCTTCAAGAGCTGTC
Fibronectin	Forward	GATGCACCGATTGTCAACAG
	Reverse	TGATCAGCATGGACCACTTC
SMA	Forward	GCAGCCATAGTTCAGAGCCTCAC
	Reverse	CATTCGTCCACCACCACCACATC
β-MHC	Forward	GCATTCTCCTGCTGTTTCCT
	Reverse	CCCAAATGCAGCCATCTC
PGC-1α	Forward	GTAAATCTGCGGGATGATGG
	Reverse	AGCAGGGTCAAAATCGTCTG
PGC-1β	Forward	TGAGGTGTTCGGTGAGATTG
	Reverse	CCATAGCTCAGGTGGAAGGA
ERRα	Forward	ATCTGCTGGTGGTTGAACCTG
	Reverse	AGAAGCCTGGGATGCTCTTG
ERRβ	Forward	CGCTTCTCTATGCAGAACCT
	Reverse	ACTTGACCATTCCTACTTCGTA
CPT-2	Forward	GTCACGGTGCAGAAAC
	Reverse	CTCTTTGTATAGACGGAGGC
ACADL	Forward	GGGAAGAGCAAGCGTACTCC
	Reverse	TCTGTCATGGCTATGGCACC
PPARγ	Forward	GAGATGCCATTCTGGCCCACCAACTTCGG
	Reverse	TATCATAAATAAGCTTCAATCGGATGGTTC

Table S1 (continued)

mRNA (mouse)		Sequence
CPT-1β	Forward	CAAGTTCAGAGACGAACGCC
	Reverse	TCAAGAGCTGTTCTCCGAACTG
PCX	Forward	GGGATGCCCACCAGTCACT
	Reverse	CATAGGGCGCAATCTTTTTGA
MCT-1	Forward	TTGTCTGTCTGGTTGCGGCTTGATCG
	Reverse	GCCCAAGACCTCCAATAACACCAATGC
PDK4	Forward	GGAAGTATCGACCCAAACTGTGA
	Reverse	GGTCGCAGAGCATCTTTGC
GPT	Forward	ACTGATGAGCGTGCGGTTGTG
	Reverse	CGTTGAAGACCTGCTCCGTGAG
GLUT4	Forward	GTAACTTCATTGTCGGCATGG
	Reverse	AGCTGAGATCTGGTCAAACG
PFKm	Forward	GCCATCGCCGTGTTGACCTC
	Reverse	GAAGATACCAACTCGGACCACAGC
SDHA	Forward	CATGCCAGGGAAGATTACAAAG
	Reverse	AGTAGGAGCGGATAGCAGGAG
ATP5A1	Forward	AATGTTCAAGCAGAGGAGATGGT
	Reverse	TCCATCAATAGCATTACCGAGGG
ATP5β	Forward	TTCAGGGGCACCAATCAAAATTC
	Reverse	CAACCTTTATCCCAGTCACCAGA
NDUS6	Forward	CAACAGCCTGTGAATGAGGTGGAG
	Reverse	TAGTGATGGTGCTGCTTGAACTGC
UQCRC1	Forward	CGCACAGATTGACTGACTACCTC
	Reverse	AGGCACGGCATCTTCTTCATACAC
GAPDH	Forward	CCATCACTGCCACTCAGAAGAC
	Reverse	TCATACTTGGCAGGTTTCTCCA

Table S2 Detailed descriptions of the detected proteins.

Antibody	Species source	Cat. No.	Source
GAPDH	Mouse	MB001	Bioworld, Bloomington, MN, USA
NDUFV2	Rabbit	15301-1-AP	Proteintech, Chicago, IL, USA
SDHB	Rabbit	10620-1-AP	Proteintech, Chicago, IL, USA
UQCRB	Rabbit	10756-1-AP	Proteintech, Chicago, IL, USA
MT-CO2	Rabbit	55070-1-AP	Proteintech, Chicago, IL, USA
ATP5D	Rabbit	14893-1-AP	Proteintech, Chicago, IL, USA
PDK4	Rabbit	12949-1-AP	Proteintech, Chicago, IL, USA
CPT-1α	Rabbit	15184-1-AP	Proteintech, Chicago, IL, USA
CD36	Rabbit	18836-1-AP	Proteintech, Chicago, IL, USA
GLUT4	Rabbit	21048-1-AP	Proteintech, Chicago, IL, USA
AMPKα	Rabbit	10929-2-AP	Proteintech, Chicago, IL, USA
PGC-1α	Rabbit	AP0774	Bioworld, Bloomington, MN, USA
PPARα	Rabbit	15540-1-AP	Proteintech, Chicago, IL, USA

Figure S1 Bar plots showing interventricular septum thickness (IVS) **(A, B)**, left ventricular diameters (LVID) **(C, D)**, and left ventricular volume (LV Vol) **(E, F)** evaluated by echocardiography in mice that underwent the sham operation, TAC only, or TAC followed by treatment with S-F or valsartan. Values shown are mean ± SD. *$p < 0.05$, **$p < 0.01$, compared with the sham group; #$p < 0.05$, ##$p < 0.01$, compared with the TAC group, analysis was performed using one-way ANOVA with Dunnett's post hoc test. d, diastolic; s, systolic.

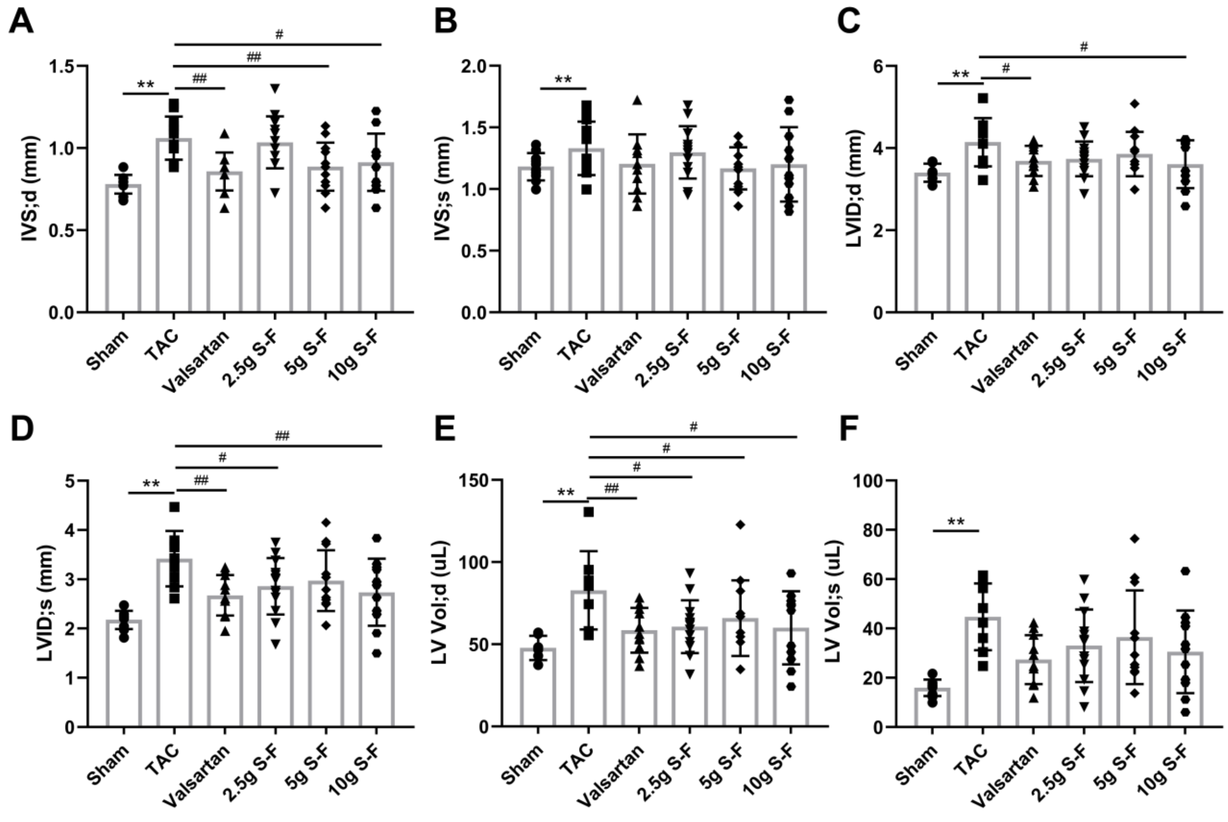

Figure S2 qRT-PCR analysis of mRNA levels of atrial natriuretic peptide (ANP) **(A)**, brain natriuretic peptide (BNP) **(B)**, collagen I **(C)**, fibronectin **(D)**, smooth muscle actin (SMA) **(E)**, α-myosin heavy chain (α-MHC) **(F)**, and β-myosin heavy chain (β-MHC) **(G)** in heart tissues. Values shown are mean ± SD. $^{*}p < 0.05$, $^{**}p < 0.01$, compared with the sham group; $^{\#}p < 0.05$, $^{\#\#}p < 0.01$, compared with the TAC group, analysis was performed using one-way ANOVA with Dunnett's post hoc test.

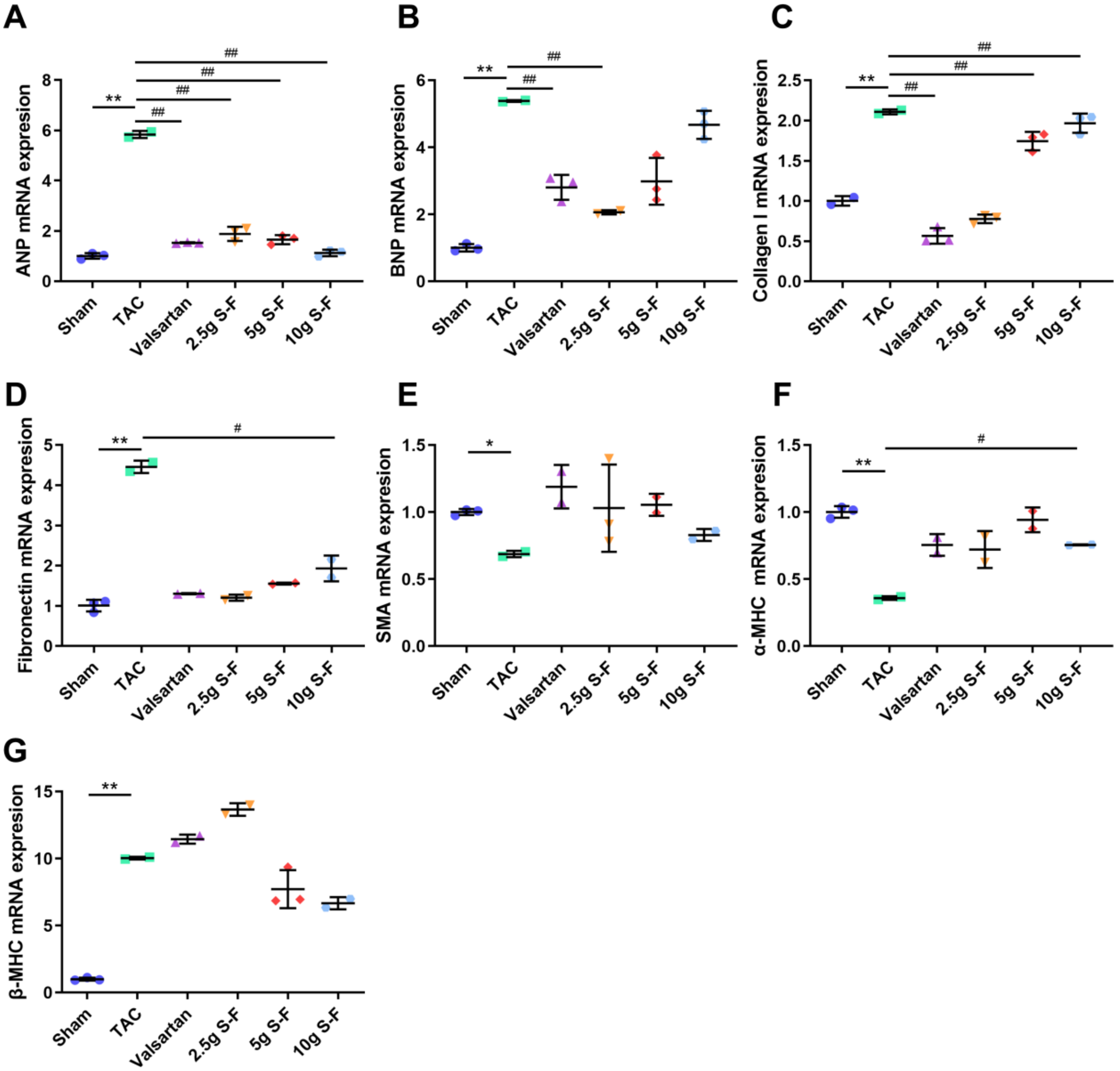

Figure S3 qRT-PCR analysis of mRNA levels of NDUS6 **(A)**, SDHA **(B)**, UQCRC1 **(C)**, ATP5A1 **(D)**, and ATP5β **(E)** in heart tissues. Values shown are mean ± SD. $^{*}p < 0.05$, $^{**}p < 0.01$, compared with the sham group; $^{\#}p < 0.05$, $^{\#\#}p < 0.01$, compared with the TAC group, analysis was performed using one-way ANOVA with Dunnett's post hoc test. NDUS6, NADH dehydrogenase ubiquinone iron-sulfur protein 6. SDHA, succinate dehydrogenase subunit A. UQCRC1, ubiquinol-cytochrome c reductase core protein 1. ATP5A1, ATP synthase α-subunit. ATP5β, ATP synthase β-subunit.

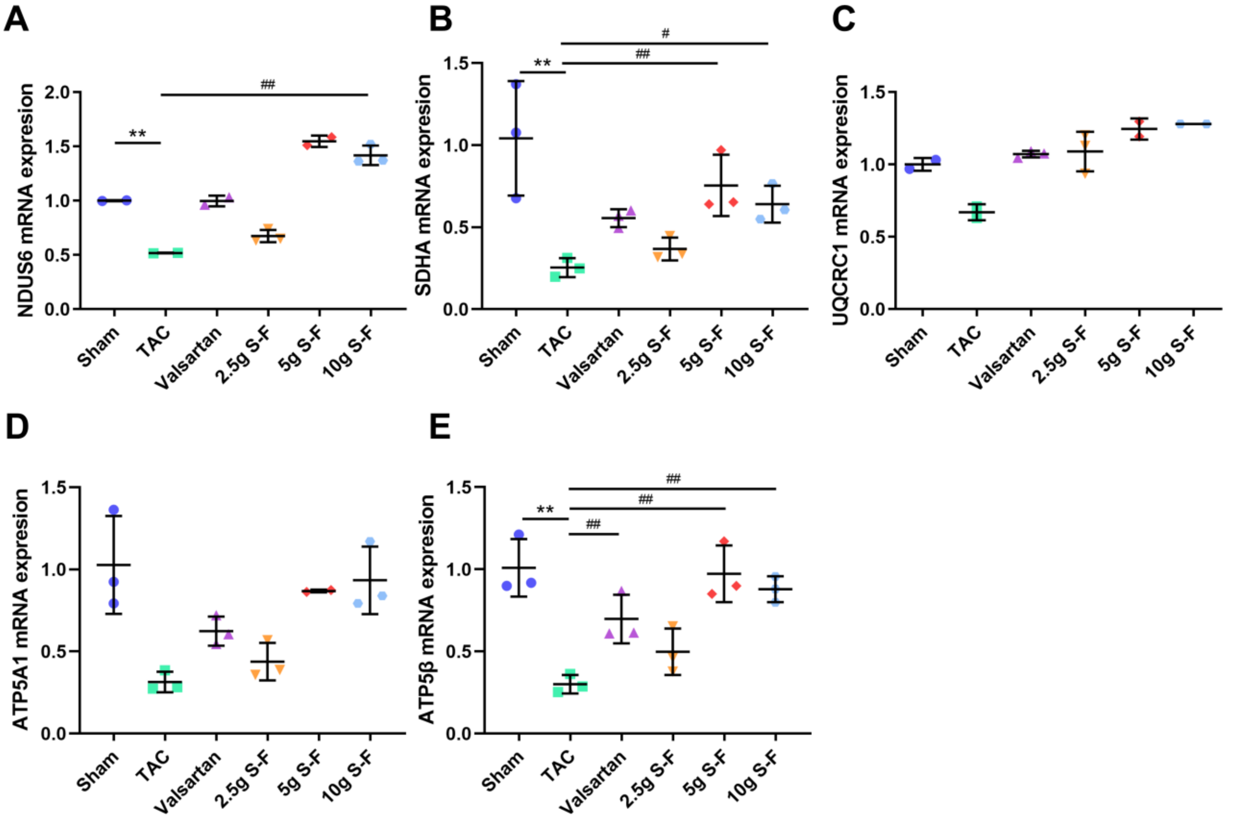

Figure S4 Transcriptomics, proteomics, and metabolomics analysis. (A) PCA scores plot of the sham, TAC and 10g S-F groups based on transcriptomics analysis. **(B, C)** Volcano plot showing the differentially expressed genes **(B)** and proteins **(C)** in the TAC group compared to the sham group. **(D, E)** PCA scores plot of the sham, TAC and 10g S-F groups derived from metabolomics analysis (**D**, negative ion mode; **E**, positive ion mode). **(F)** Venn-diagrams showing the number of differentially expressed metabolites (DEMs) in TAC mice compared to sham mice, and DEMs in S-F treated mice compared to TAC ones. **(G)** OPLS-DA scores plot derived from plasma metabolic profiling of the TAC and sham groups (positive ion mode). **(H)** OPLS-DA scores plot of the 10g S-F group versus those of the TAC group (positive ion mode). **(I)** Volcano plot showing the DEMs in the TAC group compared to the sham group. PCA, principal component analysis. OPLS-DA, orthogonal partial least squares discriminant analysis.

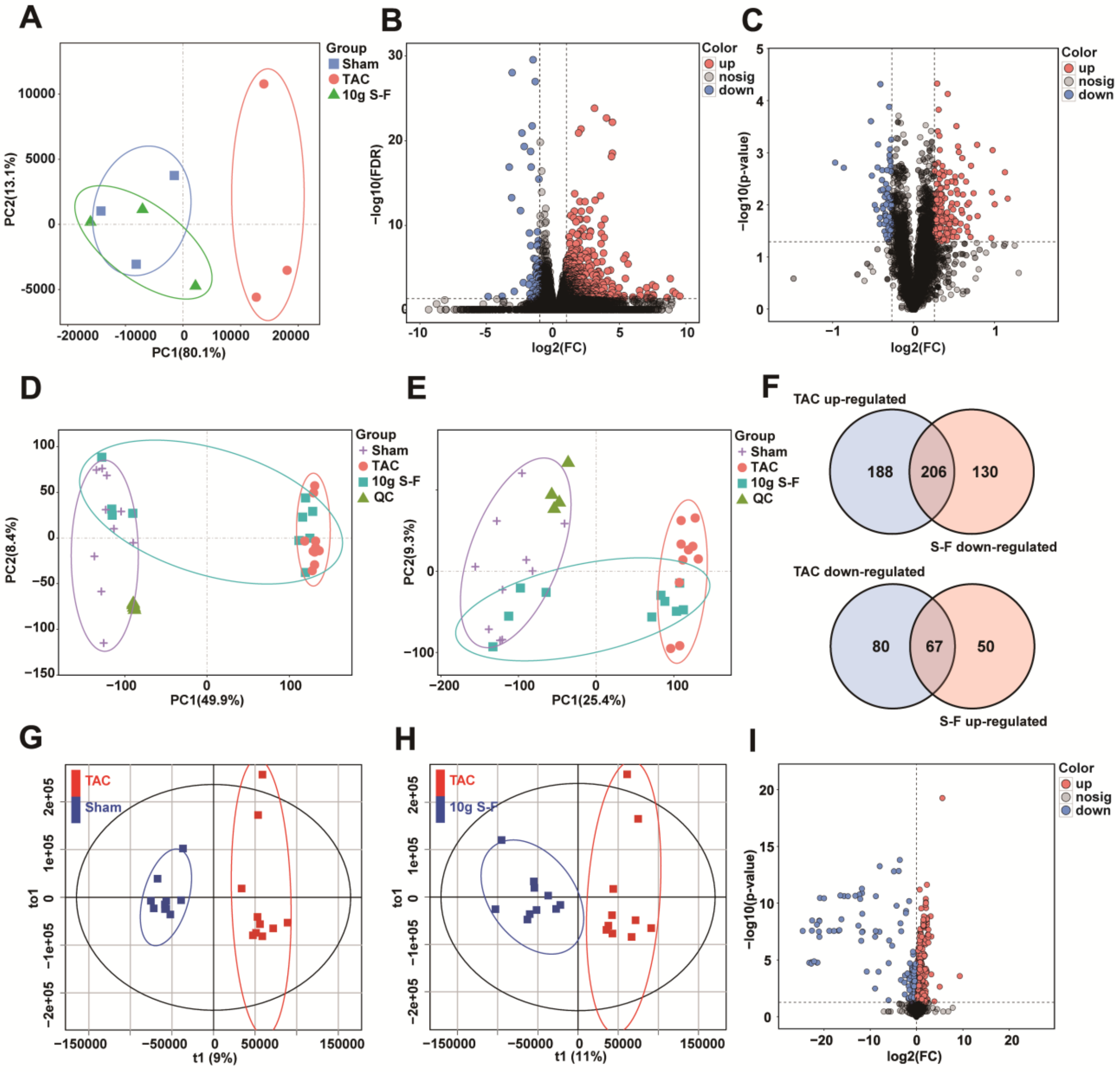
A
Group
Sham
TAC
10g S-F
PC1(80.1%)
PC2(13.1%)
B
Color
up
nosig
down
log2(FC)
−log10(FDR)
C
Color
up
nosig
down
log2(FC)
−log10(p-value)
D
Group
Sham
TAC
10g S-F
QC
PC1(49.9%)
PC2(8.4%)
E
Group
Sham
TAC
10g S-F
QC
PC1(25.4%)
PC2(9.3%)
F
TAC up-regulated
188
206
130
S-F down-regulated
TAC down-regulated
80
67
50
S-F up-regulated
G
TAC
Sham
t1 (9%)
to1
H
TAC
10g S-F
t1 (11%)
to1
I
Color
up
nosig
down
log2(FC)
−log10(p-value)

Figure S5 (A-B) qRT-PCR analysis of mRNA levels of pyruvate carboxylase (PCX) **(A)** and monocarboxylase pyruvate transporter 1 (MCT1) **(B)**. **(C)** Western blot analysis of the expression of peroxisome proliferator-activated receptor α (PPARα). Values shown are mean ± SD. *$p < 0.05$, **$p < 0.01$, compared with the sham group; $^{\#}p < 0.05$, $^{\#\#}p < 0.01$, compared with the TAC group, evaluated by one-way ANOVA with Dunnett's post hoc test.

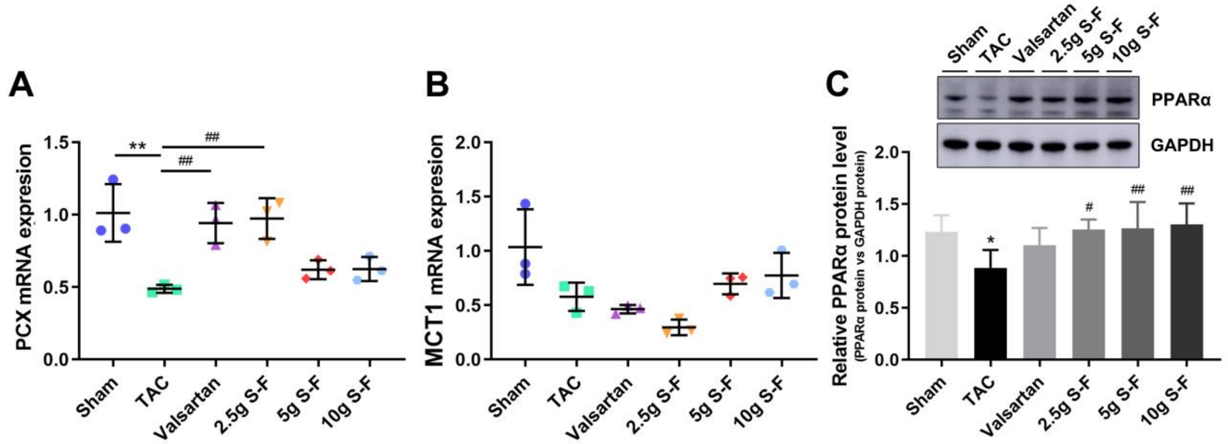

Supplementary methods

Experimental animal grouping

The whole experiment was performed in three batches of mice. The total number of animals in each group: sham group, n = 40; TAC group, n = 52; valsartan group, n = 46; 2.5g S-F group, n = 29; 5g S-F group, n = 31; 10g S-F group, n = 51. The first batch of mice was used for echocardiography evaluation, histopathological examination, western blot analysis and the measurement of myocardial extracellular matrix factors (sham group, n = 10; TAC group, n = 13; valsartan group, n = 12; 2.5g S-F group, n = 13; 5g S-F group, n = 13; 10g S-F group, n = 12). The second batch was used for the determination of HW/BW, HW/TL, LW/BW, and hemodynamic indicators, and for mitochondrial function evaluation as well as qRT-PCR analysis (sham group, n = 17; TAC group, n = 15; valsartan group, n = 13; 2.5g S-F group, n = 12; 5g S-F group, n = 13; 10g S-F group, n = 14). The third batch was used for multi-omics studies and glucose uptake measurement (sham group, n = 13; TAC group, n = 24; valsartan group, n = 21; 2.5g S-F group, n = 4; 5g S-F group, n = 5; 10g S-F group, n = 25).

Transcriptomics analysis

RNA Extraction, library construction, and sequencing

Total RNA from myocardial tissue was extracted with TRIzol reagent (Invitrogen, Carlsbad, CA, USA) for RNA-Seq and qRT-PCR. RNA quality was assessed on an Agilent 2100 Bioanalyzer (Agilent Technologies, Santa Clara, CA, USA). After total RNA was extracted, eukaryotic mRNA was enriched by Oligo(dT) beads. Then the interrupting reagent (Life Technologies, Gaithersburg, MD, USA) was added to break the mRNA into short fragments at a suitable temperature in the thermomixer. The mRNA mixtures were reverse transcribed with random primers. Second-strand cDNA was synthesized by DNA polymerase I, RNase H, dNTP, and buffer. The cDNA fragments were purified with 1.8X Agencourt AMPure XP beads (Beckman Coulter, Miami, FL, USA), end-repaired, poly(A) added, and ligated to Illumina sequencing adapters. The ligation products were amplified and sequenced using Illumina HiSeqTM 2500 (Illumina, San Diego, CA, USA).

Data processing and bioinformatics analysis

Raw data were processed using the fastp tool (version 0.18.0). Reads containing poly-Ns, duplicate sequences, and low-quality sequences were removed to obtain high-quality clean reads. Short reads alignment tool Bowtie2 (version 2.2.8) was used for mapping reads to the rRNA database, and the rRNA mapped reads were removed. The remaining reads were further mapped to the reference genome using TopHat2 (version 2.1.1). The alignment parameters were as follows: maximum read mismatch, 2; distance between mate-pair reads, 50 bp; error of distance between mate-pair reads, ±80 bp. The reconstruction of transcripts was carried out with software Cufflinks (version 2.2.1). Gene abundances were quantified by software RSEM (version 1.2.19). The FPKM (fragment per kilobase of transcript per million mapped reads) value was calculated to quantify the expression abundance and variations of each gene. Genes with the absolute fold-change greater than 1.5 and false detection rate (FDR) below 0.05 were classified as differentially expressed. Functional analysis of the differentially expressed genes was performed using the KEGG database.

Proteomics analysis

High pH Reverse Phase Separation

The peptide mixture was re-dissolved in 20 mM ammonium formate in water (pH 10.0, adjusted with ammonium hydroxide). The samples were then fractionated by high pH separation using an Ultimate 3000 system (Thermo Fisher Scientific, Waltham, MA, USA). The column was a 4.6 mm × 250 mm, 5 µm, XBridge C18 reverse phase column (Waters Corporation, Milford, MA, USA) and the temperature was maintained at 30°C. The mobile phase consisted of 20 mM ammonium formate in water (pH 10.0, adjusted with ammonium hydroxide, mobile phase A) and 20 mM ammonium formate in 80% CAN (pH 10.0, adjusted with ammonium hydroxide, mobile phase B). The linear gradient was starting from 5% B to 45% B in 40 min with a flow rate of 1 ml/min. A total of 12 fractions were collected, each fraction was dried in a vacuum concentrator for the next step.

Low pH nano-HPLC Orbitrap-MS/MS analysis

Peptide fractions were reconstituted with 30 µl of 0.1% formic acid in water. The samples were further analyzed on an Easy-nLC 1000 system connected to a Q Exactive Hybrid Quadrupole-Orbitrap system (Thermo Fisher Scientific, Waltham, MA, USA). After loading onto an Acclaim PepMap C18 100 µm × 2 cm trap column at a flow rate of 10 µl/min for 3 min, samples were applied onto an Acclaim PepMap C18 75 µm × 15 cm analytical column. The column flow rate was 300 nl/min, and the temperature was maintained at 40°C. The mobile phase consisted of 0.1% formic acid in water (mobile phase A) and 0.1% formic acid in acetonitrile (mobile phase B) with a linear gradient, from 2% B to 40% B in 70 min. The Q Exactive mass spectrometer was operated in the data-dependent acquisition mode to switch automatically between MS and MS/MS acquisition. The following program settings were applied: m/z scan range, 350-1550 Da; electrospray voltage, 2 kV; full MS resolution, 70000; MS/MS resolution, 17500; collision energy as 27 eV in NCE model.

Data processing and quantitative analysis

For protein identification and quantification, the raw data of mass spectra were extracted, charge state deconvoluted and deisotoped by Mascot Distiller version 2.6 (Matrix Science, London, UK). The processed data were then transformed into MGF format by Proteome Discovery 1.2 (Thermo Fisher Scientific, Waltham, MA, USA). MGF files were searched using the Mascot search engine (version 2.3.2, Matrix Science, London, UK). The search parameters were as follows: enzyme, trypsin; one missed cleavages allowed; taxonomy, Mus musculus; peptide mass tolerance, 20 ppm; and fragment mass tolerance, 0.05 Da.

Protein identifications were accepted if they could achieve an FDR less than 1.0% by the Scaffold Local FDR algorithm. Confident protein identification involved at least two unique peptides. Protein relative quantification was based on the ratios of reporter ions, which reflect the relative abundance of peptides. The Mascot search results were averaged using medians and quantified. Proteins with fold change in a comparison > 1.2 or < 0.83 and unadjusted significance level $p < 0.05$ were considered differentially expressed.

Metabolomics analysis

UHPLC Q-Orbitrap-MS/MS experimental parameters in the metabolomic study

The metabolomic analysis was characterized using a UHPLC system (1290, Agilent Technologies, Santa Clara, CA, USA) with a UPLC HSS T3 column (2.1 mm × 100 mm, 1.8 μm) coupled to a Q Exactive Hybrid Quadrupole-Orbitrap system (Thermo Fisher Scientific, Waltham, MA, USA). The mobile phase A was 0.1% formic acid in water for positive ion mode, and 5 mmol/l ammonium acetate in water for negative, and the mobile phase B was acetonitrile. The elution gradient was set as follows: 0-1 min, 1% B; 1-8 min, 99%-1% B; 8-10 min, 1% B. The flow rate was 0.5 ml/min. The injection volume was 2 μl. The mass-spectrometric data were collected in full scan mode from m/z 70 to 1000 under the following conditions: spray voltage, 3.8 kV (positive ion mode) and -3.1 kV (negative ion mode); sheath gas flow rate, 45 Arb; auxiliary gas flow rate, 15 Arb; capillary temperature, 320°C; full MS resolution, 70000; MS/MS resolution, 17500; collision energy as 20/40/60 eV in NCE model.

In order to test the repeatability of the analytical system, the QC samples were injected after every ten samples throughout the analytical workflow. Base peak chromatograms and PCA scores plots demonstrated good technical repeatability from QC samples, indicating that the analysis system was stable and all the data were under control.

Data processing and multivariate analysis

MS raw data (.raw) files were converted to the mzML format using ProteoWizard and processed by R package XCMS, including retention time alignment, peak detection and peak matching. Then data filtering was carried out, including estimating missing values, data filtering, data normalization and Pareto scaling. The processed data were further imported into the SIMCA 14.1 software package (Umetrics, Umeå, Sweden) for multivariate data analysis. Principal component analysis (PCA) and orthogonal partial least squares discriminant analysis (OPLS-DA) were performed to maximize class differences while minimizing the variability unrelated to class. The goodness-of-fit was quantified by R^2, while the predictive ability was indicated by Q^2. A cross-

validation procedure and testing with 200 random permutations were performed to avoid over-fitting of supervised OPLS-DA models. Furthermore, potential biomarkers were selected based on the variable importance of project (VIP) statistics and the two-tailed p-values calculated by Student's t-test. The metabolites were identified by matching the accurate mass of observed peaks with those in the HMDB and KEGG databases within a mass accuracy window of 10 ppm.
